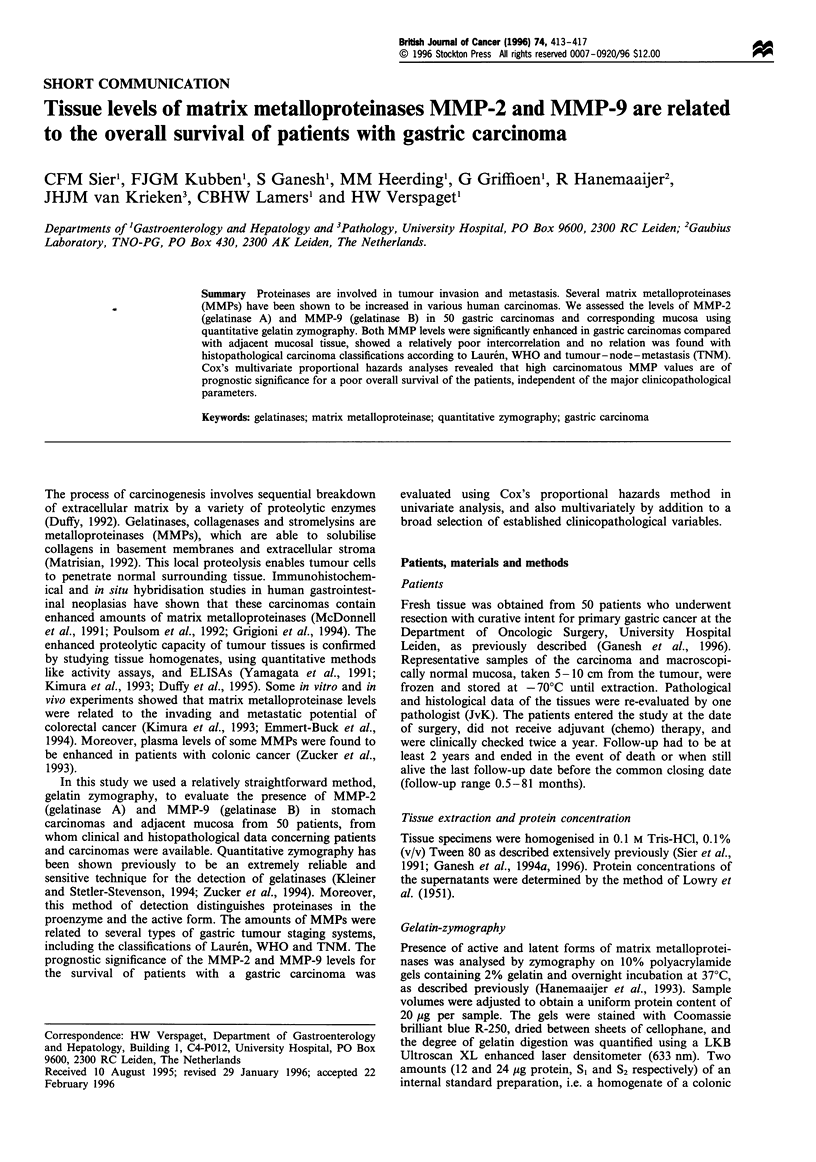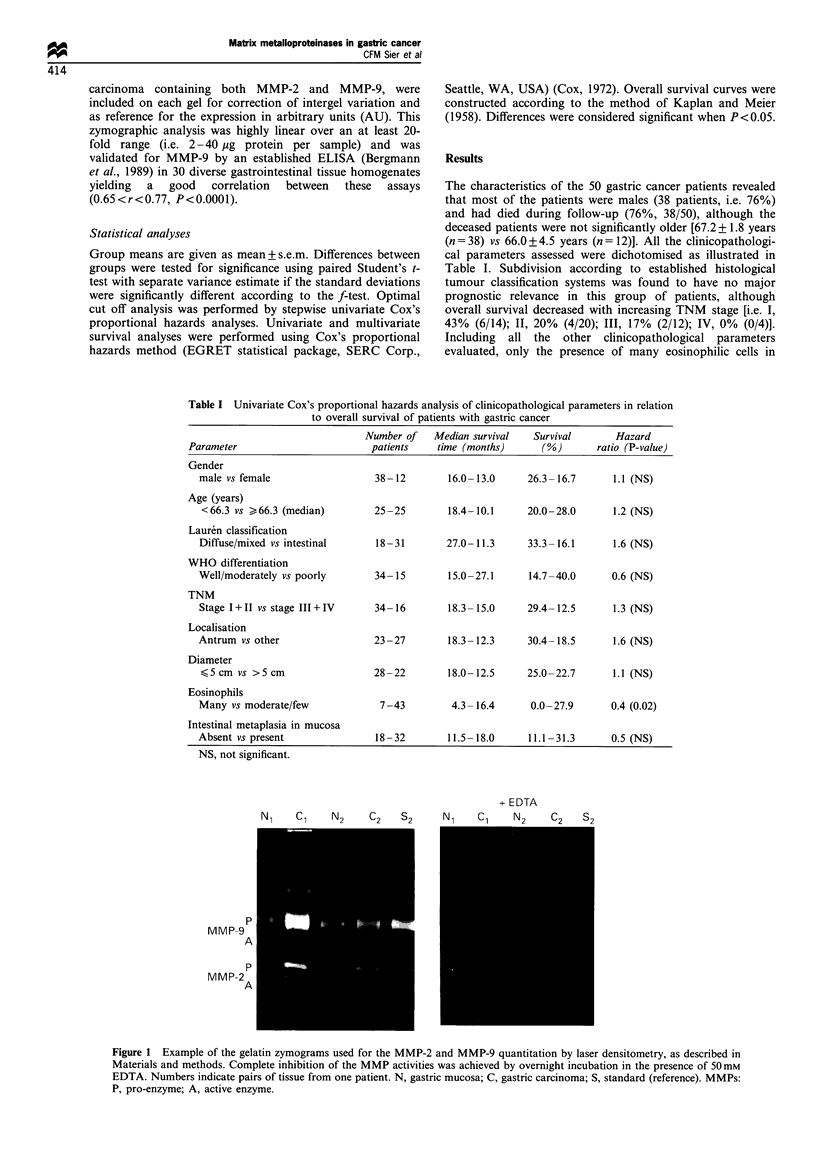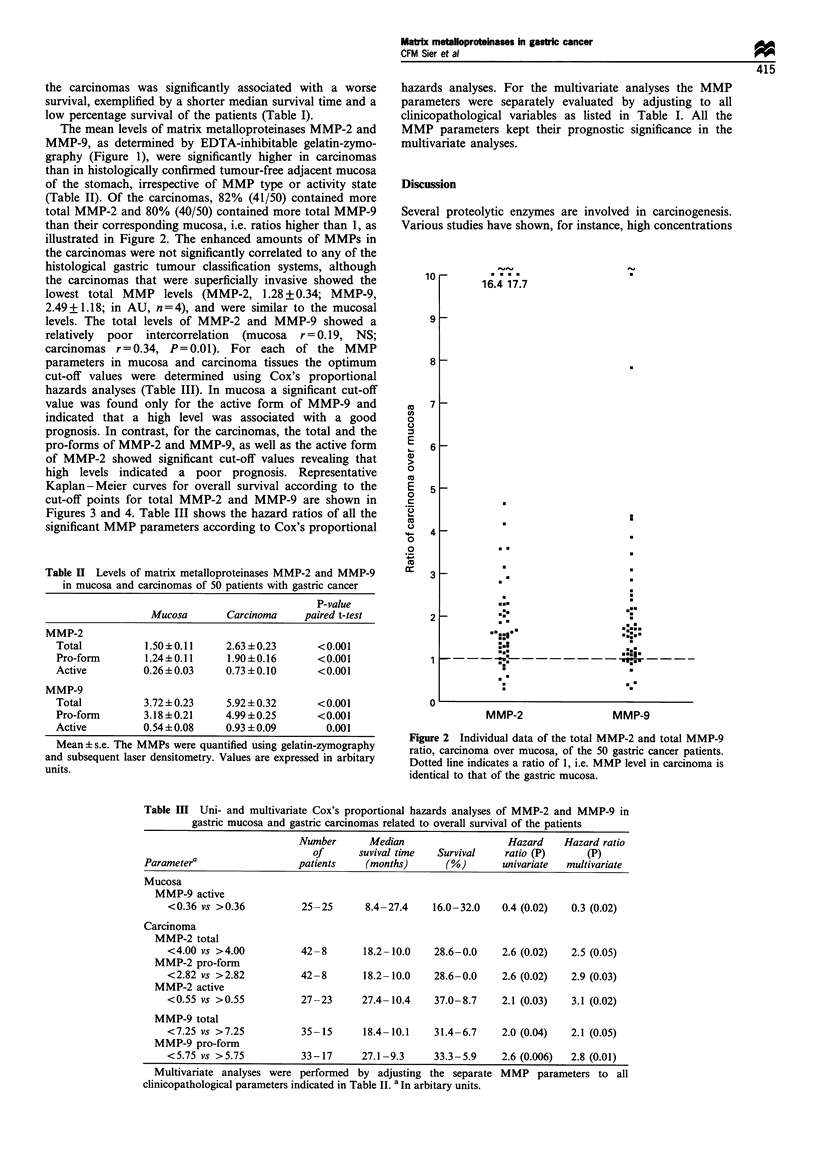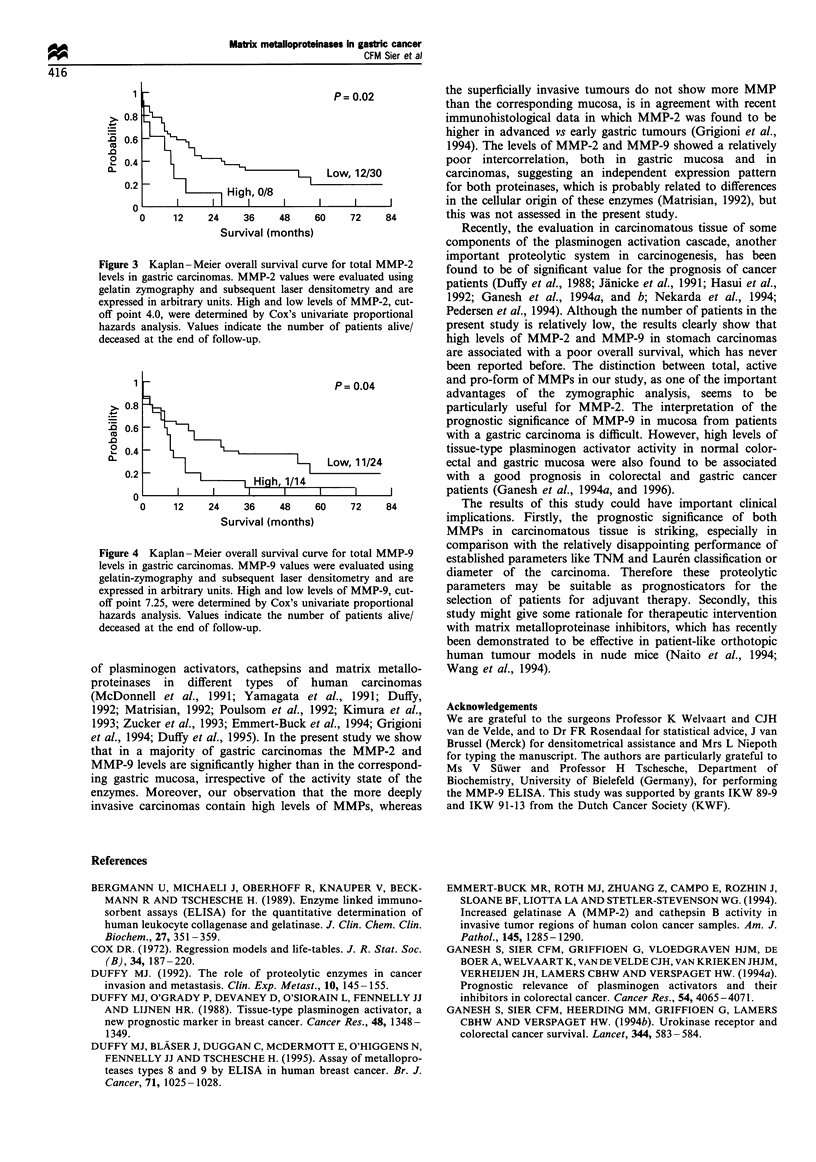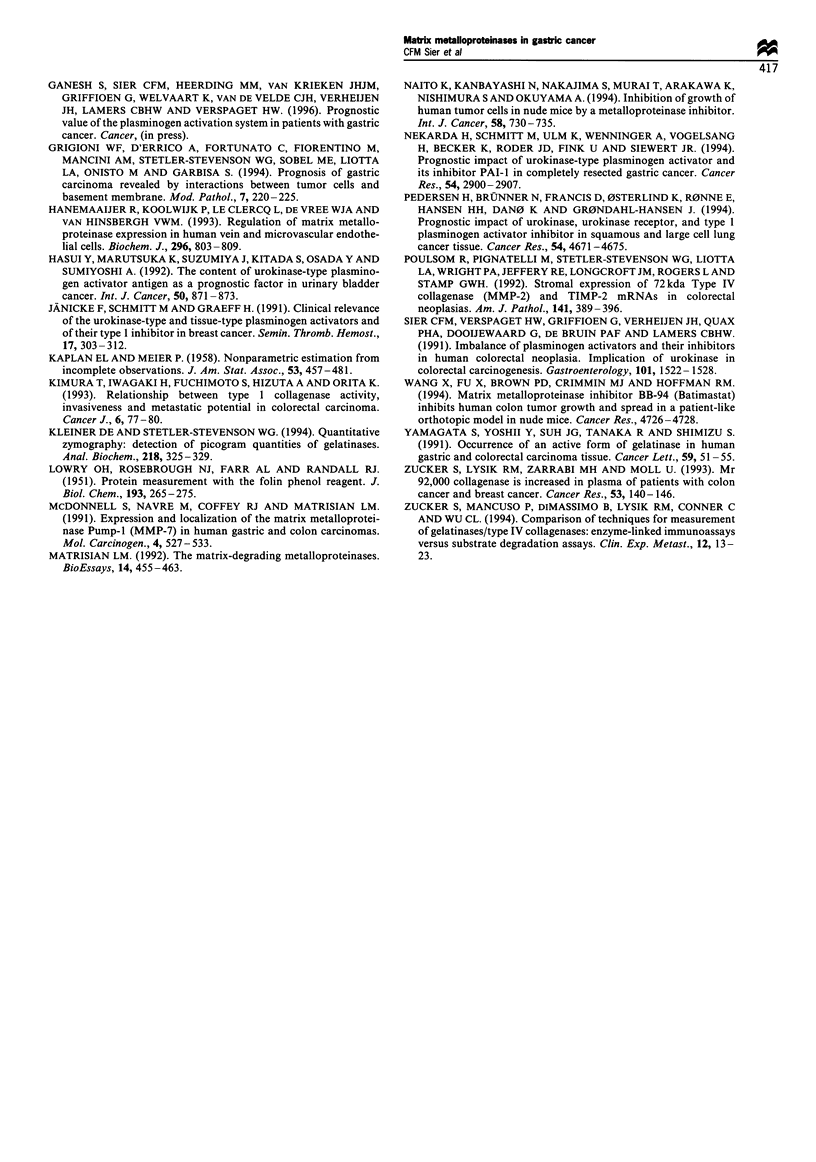# Tissue levels of matrix metalloproteinases MMP-2 and MMP-9 are related to the overall survival of patients with gastric carcinoma.

**DOI:** 10.1038/bjc.1996.374

**Published:** 1996-08

**Authors:** C. F. Sier, F. J. Kubben, S. Ganesh, M. M. Heerding, G. Griffioen, R. Hanemaaijer, J. H. van Krieken, C. B. Lamers, H. W. Verspaget

**Affiliations:** Department of Gastroenterology and Hepatology, University Hospital, Leiden, The Netherlands.

## Abstract

**Images:**


British Journal of Cancer (1996) 74, 413-417
© 1996 Stockton Press All rights reserved 0007-0920/96 $12.00

SHORT COMMUNICATION

### Tissue levels of matrix metalloproteinases MMP-2 and MMP-9 are related to the overall survival of patients with gastric carcinoma

CFM Sier[1], FJGM Kubben[1], S Ganesh[1], MM Heerding[1], G Griffioen[1], R Hanemaaijer[2], JHJM van Krieken[3], CBHW Lamers[1] and HW Verspaget[1]

*Departments of [1]Gastroenterology and Hepatology and [3]Pathology, University Hospital, PO Box 9600, 2300 RC Leiden; [2]Gaubius Laboratory, TNO-PG, PO Box 430, 2300 AK Leiden, The Netherlands.*

**Summary** Proteinases are involved in tumour invasion and metastasis. Several matrix metalloproteinases (MMPs) have been shown to be increased in various human carcinomas. We assessed the levels of MMP-2 (gelatinase A) and MMP-9 (gelatinase B) in 50 gastric carcinomas and corresponding mucosa using quantitative gelatin zymography. Both MMP levels were significantly enhanced in gastric carcinomas compared with adjacent mucosal tissue, showed a relatively poor intercorrelation and no relation was found with histopathological carcinoma classifications according to Laurén, WHO and tumour-node-metastasis (TNM). Cox's multivariate proportional hazards analyses revealed that high carcinomatous MMP values are of prognostic significance for a poor overall survival of the patients, independent of the major clinicopathological parameters.

**Keywords:** gelatinases; matrix metalloproteinase; quantitative zymography; gastric carcinoma

The process of carcinogenesis involves sequential breakdown of extracellular matrix by a variety of proteolytic enzymes (Duffy, 1992). Gelatinases, collagenases and stromelysins are metalloproteinases (MMPs), which are able to solubilise collagens in basement membranes and extracellular stroma (Matrisian, 1992). This local proteolysis enables tumour cells to penetrate normal surrounding tissue. Immunohistochemical and *in situ* hybridisation studies in human gastrointestinal neoplasias have shown that these carcinomas contain enhanced amounts of matrix metalloproteinases (McDonnell *et al.*, 1991; Poulsom *et al.*, 1992; Grigioni *et al.*, 1994). The enhanced proteolytic capacity of tumour tissues is confirmed by studying tissue homogenates, using quantitative methods like activity assays, and ELISAs (Yamagata *et al.*, 1991; Kimura *et al.*, 1993; Duffy *et al.*, 1995). Some *in vitro* and *in vivo* experiments showed that matrix metalloproteinase levels were related to the invading and metastatic potential of colorectal cancer (Kimura *et al.*, 1993; Emmert-Buck *et al.*, 1994). Moreover, plasma levels of some MMPs were found to be enhanced in patients with colonic cancer (Zucker *et al.*, 1993).

In this study we used a relatively straightforward method, gelatin zymography, to evaluate the presence of MMP-2 (gelatinase A) and MMP-9 (gelatinase B) in stomach carcinomas and adjacent mucosa from 50 patients, from whom clinical and histopathological data concerning patients and carcinomas were available. Quantitative zymography has been shown previously to be an extremely reliable and sensitive technique for the detection of gelatinases (Kleiner and Stetler-Stevenson, 1994; Zucker *et al.*, 1994). Moreover, this method of detection distinguishes proteinases in the proenzyme and the active form. The amounts of MMPs were related to several types of gastric tumour staging systems, including the classifications of Laurén, WHO and TNM. The prognostic significance of the MMP-2 and MMP-9 levels for the survival of patients with a gastric carcinoma was evaluated using Cox's proportional hazards method in univariate analysis, and also multivariately by addition to a broad selection of established clinicopathological variables.

### Patients, materials and methods

### *Patients*

Fresh tissue was obtained from 50 patients who underwent resection with curative intent for primary gastric cancer at the Department of Oncologic Surgery, University Hospital Leiden, as previously described (Ganesh *et al.*, 1996). Representative samples of the carcinoma and macroscopically normal mucosa, taken 5-10 cm from the tumour, were frozen and stored at $-70°C$ until extraction. Pathological and histological data of the tissues were re-evaluated by one pathologist (JvK). The patients entered the study at the date of surgery, did not receive adjuvant (chemo) therapy, and were clinically checked twice a year. Follow-up had to be at least 2 years and ended in the event of death or when still alive the last follow-up date before the common closing date (follow-up range 0.5-81 months).

### *Tissue extraction and protein concentration*

Tissue specimens were homogenised in 0.1 M Tris-HCl, 0.1% (v/v) Tween 80 as described extensively previously (Sier *et al.*, 1991; Ganesh *et al.*, 1994*a*, 1996). Protein concentrations of the supernatants were determined by the method of Lowry *et al.* (1951).

### *Gelatin-zymography*

Presence of active and latent forms of matrix metalloproteinases was analysed by zymography on 10% polyacrylamide gels containing 2% gelatin and overnight incubation at 37°C, as described previously (Hanemaaijer *et al.*, 1993). Sample volumes were adjusted to obtain a uniform protein content of 20 $\mu g$ per sample. The gels were stained with Coomassie brilliant blue R-250, dried between sheets of cellophane, and the degree of gelatin digestion was quantified using a LKB Ultroscan XL enhanced laser densitometer (633 nm). Two amounts (12 and 24 $\mu g$ protein, $S_1$ and $S_2$ respectively) of an internal standard preparation, i.e. a homogenate of a colonic

Correspondence: HW Verspaget, Department of Gastroenterology and Hepatology, Building 1, C4-P012, University Hospital, PO Box 9600, 2300 RC Leiden, The Netherlands
Received 10 August 1995; revised 29 January 1996; accepted 22 February 1996

carcinoma containing both MMP-2 and MMP-9, were included on each gel for correction of intergel variation and as reference for the expression in arbitrary units (AU). This zymographic analysis was highly linear over an at least 20-fold range (i.e. 2–40 μg protein per sample) and was validated for MMP-9 by an established ELISA (Bergmann *et al.*, 1989) in 30 diverse gastrointestinal tissue homogenates yielding a good correlation between these assays ($0.65 < r < 0.77$, $P < 0.0001$).

### *Statistical analyses*

Group means are given as mean ± s.e.m. Differences between groups were tested for significance using paired Student's *t*-test with separate variance estimate if the standard deviations were significantly different according to the *f*-test. Optimal cut off analysis was performed by stepwise univariate Cox's proportional hazards analyses. Univariate and multivariate survival analyses were performed using Cox's proportional hazards method (EGRET statistical package, SERC Corp., Seattle, WA, USA) (Cox, 1972). Overall survival curves were constructed according to the method of Kaplan and Meier (1958). Differences were considered significant when $P < 0.05$.

### Results

The characteristics of the 50 gastric cancer patients revealed that most of the patients were males (38 patients, i.e. 76%) and had died during follow-up (76%, 38/50), although the deceased patients were not significantly older [67.2 ± 1.8 years ($n = 38$) *vs* 66.0 ± 4.5 years ($n = 12$)]. All the clinicopathological parameters assessed were dichotomised as illustrated in Table I. Subdivision according to established histological tumour classification systems was found to have no major prognostic relevance in this group of patients, although overall survival decreased with increasing TNM stage [i.e. I, 43% (6/14); II, 20% (4/20); III, 17% (2/12); IV, 0% (0/4)]. Including all the other clinicopathological parameters evaluated, only the presence of many eosinophilic cells in

**Table I** Univariate Cox's proportional hazards analysis of clinicopathological parameters in relation to overall survival of patients with gastric cancer

| *Parameter* | *Number of patients* | *Median survival time (months)* | *Survival (%)* | *Hazard ratio (P-value)* |
|---|---|---|---|---|
| Gender | | | | |
| male *vs* female | 38–12 | 16.0–13.0 | 26.3–16.7 | 1.1 (NS) |
| Age (years) | | | | |
| <66.3 *vs* ⩾66.3 (median) | 25–25 | 18.4–10.1 | 20.0–28.0 | 1.2 (NS) |
| Laurén classification | | | | |
| Diffuse/mixed *vs* intestinal | 18–31 | 27.0–11.3 | 33.3–16.1 | 1.6 (NS) |
| WHO differentiation | | | | |
| Well/moderately *vs* poorly | 34–15 | 15.0–27.1 | 14.7–40.0 | 0.6 (NS) |
| TNM | | | | |
| Stage I+II *vs* stage III+IV | 34–16 | 18.3–15.0 | 29.4–12.5 | 1.3 (NS) |
| Localisation | | | | |
| Antrum *vs* other | 23–27 | 18.3–12.3 | 30.4–18.5 | 1.6 (NS) |
| Diameter | | | | |
| ⩽5 cm *vs* >5 cm | 28–22 | 18.0–12.5 | 25.0–22.7 | 1.1 (NS) |
| Eosinophils | | | | |
| Many *vs* moderate/few | 7–43 | 4.3–16.4 | 0.0–27.9 | 0.4 (0.02) |
| Intestinal metaplasia in mucosa | | | | |
| Absent *vs* present | 18–32 | 11.5–18.0 | 11.1–31.3 | 0.5 (NS) |

NS, not significant.

**Figure 1** Example of the gelatin zymograms used for the MMP-2 and MMP-9 quantitation by laser densitometry, as described in Materials and methods. Complete inhibition of the MMP activities was achieved by overnight incubation in the presence of 50 mM EDTA. Numbers indicate pairs of tissue from one patient. N, gastric mucosa; C, gastric carcinoma; S, standard (reference). MMPs: P, pro-enzyme; A, active enzyme.

the carcinomas was significantly associated with a worse survival, exemplified by a shorter median survival time and a low percentage survival of the patients (Table I).

The mean levels of matrix metalloproteinases MMP-2 and MMP-9, as determined by EDTA-inhibitable gelatin-zymography (Figure 1), were significantly higher in carcinomas than in histologically confirmed tumour-free adjacent mucosa of the stomach, irrespective of MMP type or activity state (Table II). Of the carcinomas, 82% (41/50) contained more total MMP-2 and 80% (40/50) contained more total MMP-9 than their corresponding mucosa, i.e. ratios higher than 1, as illustrated in Figure 2. The enhanced amounts of MMPs in the carcinomas were not significantly correlated to any of the histological gastric tumour classification systems, although the carcinomas that were superficially invasive showed the lowest total MMP levels (MMP-2, 1.28±0.34; MMP-9, 2.49±1.18; in AU, $n=4$), and were similar to the mucosal levels. The total levels of MMP-2 and MMP-9 showed a relatively poor intercorrelation (mucosa $r=0.19$, NS; carcinomas $r=0.34$, $P=0.01$). For each of the MMP parameters in mucosa and carcinoma tissues the optimum cut-off values were determined using Cox's proportional hazards analyses (Table III). In mucosa a significant cut-off value was found only for the active form of MMP-9 and indicated that a high level was associated with a good prognosis. In contrast, for the carcinomas, the total and the pro-forms of MMP-2 and MMP-9, as well as the active form of MMP-2 showed significant cut-off values revealing that high levels indicated a poor prognosis. Representative Kaplan–Meier curves for overall survival according to the cut-off points for total MMP-2 and MMP-9 are shown in Figures 3 and 4. Table III shows the hazard ratios of all the significant MMP parameters according to Cox's proportional hazards analyses. For the multivariate analyses the MMP parameters were separately evaluated by adjusting to all clinicopathological variables as listed in Table I. All the MMP parameters kept their prognostic significance in the multivariate analyses.

**Table II** Levels of matrix metalloproteinases MMP-2 and MMP-9 in mucosa and carcinomas of 50 patients with gastric cancer

| | *Mucosa* | *Carcinoma* | P-*value* *paired* t-*test* |
|---|---|---|---|
| MMP-2 | | | |
| Total | 1.50±0.11 | 2.63±0.23 | <0.001 |
| Pro-form | 1.24±0.11 | 1.90±0.16 | <0.001 |
| Active | 0.26±0.03 | 0.73±0.10 | <0.001 |
| MMP-9 | | | |
| Total | 3.72±0.23 | 5.92±0.32 | <0.001 |
| Pro-form | 3.18±0.21 | 4.99±0.25 | <0.001 |
| Active | 0.54±0.08 | 0.93±0.09 | 0.001 |

Mean±s.e. The MMPs were quantified using gelatin-zymography and subsequent laser densitometry. Values are expressed in arbitary units.

### Discussion

Several proteolytic enzymes are involved in carcinogenesis. Various studies have shown, for instance, high concentrations

**Figure 2** Individual data of the total MMP-2 and total MMP-9 ratio, carcinoma over mucosa, of the 50 gastric cancer patients. Dotted line indicates a ratio of 1, i.e. MMP level in carcinoma is identical to that of the gastric mucosa.

**Table III** Uni- and multivariate Cox's proportional hazards analyses of MMP-2 and MMP-9 in gastric mucosa and gastric carcinomas related to overall survival of the patients

| *Parameter*[a] | *Number of patients* | *Median suvival time (months)* | *Survival (%)* | *Hazard ratio* (P) *univariate* | *Hazard ratio* (P) *multivariate* |
|---|---|---|---|---|---|
| Mucosa | | | | | |
| MMP-9 active | | | | | |
| <0.36 *vs* >0.36 | 25–25 | 8.4–27.4 | 16.0–32.0 | 0.4 (0.02) | 0.3 (0.02) |
| Carcinoma | | | | | |
| MMP-2 total | | | | | |
| <4.00 *vs* >4.00 | 42–8 | 18.2–10.0 | 28.6–0.0 | 2.6 (0.02) | 2.5 (0.05) |
| MMP-2 pro-form | | | | | |
| <2.82 *vs* >2.82 | 42–8 | 18.2–10.0 | 28.6–0.0 | 2.6 (0.02) | 2.9 (0.03) |
| MMP-2 active | | | | | |
| <0.55 *vs* >0.55 | 27–23 | 27.4–10.4 | 37.0–8.7 | 2.1 (0.03) | 3.1 (0.02) |
| MMP-9 total | | | | | |
| <7.25 *vs* >7.25 | 35–15 | 18.4–10.1 | 31.4–6.7 | 2.0 (0.04) | 2.1 (0.05) |
| MMP-9 pro-form | | | | | |
| <5.75 *vs* >5.75 | 33–17 | 27.1–9.3 | 33.3–5.9 | 2.6 (0.006) | 2.8 (0.01) |

Multivariate analyses were performed by adjusting the separate MMP parameters to all clinicopathological parameters indicated in Table II. [a] In arbitary units.

**Figure 3** Kaplan–Meier overall survival curve for total MMP-2 levels in gastric carcinomas. MMP-2 values were evaluated using gelatin zymography and subsequent laser densitometry and are expressed in arbitrary units. High and low levels of MMP-2, cut-off point 4.0, were determined by Cox's univariate proportional hazards analysis. Values indicate the number of patients alive/deceased at the end of follow-up.

**Figure 4** Kaplan–Meier overall survival curve for total MMP-9 levels in gastric carcinomas. MMP-9 values were evaluated using gelatin-zymography and subsequent laser densitometry and are expressed in arbitrary units. High and low levels of MMP-9, cut-off point 7.25, were determined by Cox's univariate proportional hazards analysis. Values indicate the number of patients alive/deceased at the end of follow-up.

of plasminogen activators, cathepsins and matrix metalloproteinases in different types of human carcinomas (McDonnell *et al.*, 1991; Yamagata *et al.*, 1991; Duffy, 1992; Matrisian, 1992; Poulsom *et al.*, 1992; Kimura *et al.*, 1993; Zucker *et al.*, 1993; Emmert-Buck *et al.*, 1994; Grigioni *et al.*, 1994; Duffy *et al.*, 1995). In the present study we show that in a majority of gastric carcinomas the MMP-2 and MMP-9 levels are significantly higher than in the corresponding gastric mucosa, irrespective of the activity state of the enzymes. Moreover, our observation that the more deeply invasive carcinomas contain high levels of MMPs, whereas the superficially invasive tumours do not show more MMP than the corresponding mucosa, is in agreement with recent immunohistological data in which MMP-2 was found to be higher in advanced *vs* early gastric tumours (Grigioni *et al.*, 1994). The levels of MMP-2 and MMP-9 showed a relatively poor intercorrelation, both in gastric mucosa and in carcinomas, suggesting an independent expression pattern for both proteinases, which is probably related to differences in the cellular origin of these enzymes (Matrisian, 1992), but this was not assessed in the present study.

Recently, the evaluation in carcinomatous tissue of some components of the plasminogen activation cascade, another important proteolytic system in carcinogenesis, has been found to be of significant value for the prognosis of cancer patients (Duffy *et al.*, 1988; Jänicke *et al.*, 1991; Hasui *et al.*, 1992; Ganesh *et al.*, 1994*a*, and *b*; Nekarda *et al.*, 1994; Pedersen *et al.*, 1994). Although the number of patients in the present study is relatively low, the results clearly show that high levels of MMP-2 and MMP-9 in stomach carcinomas are associated with a poor overall survival, which has never been reported before. The distinction between total, active and pro-form of MMPs in our study, as one of the important advantages of the zymographic analysis, seems to be particularly useful for MMP-2. The interpretation of the prognostic significance of MMP-9 in mucosa from patients with a gastric carcinoma is difficult. However, high levels of tissue-type plasminogen activator activity in normal colorectal and gastric mucosa were also found to be associated with a good prognosis in colorectal and gastric cancer patients (Ganesh *et al.*, 1994*a*, and 1996).

The results of this study could have important clinical implications. Firstly, the prognostic significance of both MMPs in carcinomatous tissue is striking, especially in comparison with the relatively disappointing performance of established parameters like TNM and Laurén classification or diameter of the carcinoma. Therefore these proteolytic parameters may be suitable as prognosticators for the selection of patients for adjuvant therapy. Secondly, this study might give some rationale for therapeutic intervention with matrix metalloproteinase inhibitors, which has recently been demonstrated to be effective in patient-like orthotopic human tumour models in nude mice (Naito *et al.*, 1994; Wang *et al.*, 1994).

**Acknowledgements**

We are grateful to the surgeons Professor K Welvaart and CJH van de Velde, and to Dr FR Rosendaal for statistical advice, J van Brussel (Merck) for densitometrical assistance and Mrs L Niepoth for typing the manuscript. The authors are particularly grateful to Ms V Süwer and Professor H Tschesche, Department of Biochemistry, University of Bielefeld (Germany), for performing the MMP-9 ELISA. This study was supported by grants IKW 89-9 and IKW 91-13 from the Dutch Cancer Society (KWF).

### References

BERGMANN U, MICHAELI J, OBERHOFF R, KNAUPER V, BECKMANN R AND TSCHESCHE H. (1989). Enzyme linked immunosorbent assays (ELISA) for the quantitative determination of human leukocyte collagenase and gelatinase. *J. Clin. Chem. Clin. Biochem.*, **27**, 351–359.

COX DR. (1972). Regression models and life-tables. *J. R. Stat. Soc. (B)*, **34**, 187–220.

DUFFY MJ. (1992). The role of proteolytic enzymes in cancer invasion and metastasis. *Clin. Exp. Metast.*, **10**, 145–155.

DUFFY MJ, O'GRADY P, DEVANEY D, O'SIORAIN L, FENNELLY JJ AND LIJNEN HR. (1988). Tissue-type plasminogen activator, a new prognostic marker in breast cancer. *Cancer Res.*, **48**, 1348–1349.

DUFFY MJ, BLÄSER J, DUGGAN C, MCDERMOTT E, O'HIGGENS N, FENNELLY JJ AND TSCHESCHE H. (1995). Assay of metalloproteases types 8 and 9 by ELISA in human breast cancer. *Br. J. Cancer*, **71**, 1025–1028.

EMMERT-BUCK MR, ROTH MJ, ZHUANG Z, CAMPO E, ROZHIN J, SLOANE BF, LIOTTA LA AND STETLER-STEVENSON WG. (1994). Increased gelatinase A (MMP-2) and cathepsin B activity in invasive tumor regions of human colon cancer samples. *Am. J. Pathol.*, **145**, 1285–1290.

GANESH S, SIER CFM, GRIFFIOEN G, VLOEDGRAVEN HJM, DE BOER A, WELVAART K, VAN DE VELDE CJH, VAN KRIEKEN JHJM, VERHEIJEN JH, LAMERS CBHW AND VERSPAGET HW. (1994*a*). Prognostic relevance of plasminogen activators and their inhibitors in colorectal cancer. *Cancer Res.*, **54**, 4065–4071.

GANESH S, SIER CFM, HEERDING MM, GRIFFIOEN G, LAMERS CBHW AND VERSPAGET HW. (1994*b*). Urokinase receptor and colorectal cancer survival. *Lancet*, **344**, 583–584.

GANESH S, SIER CFM, HEERDING MM, VAN KRIEKEN JHJM, GRIFFIOEN G, WELVAART K, VAN DE VELDE CJH, VERHEIJEN JH, LAMERS CBHW AND VERSPAGET HW. (1996). Prognostic value of the plasminogen activation system in patients with gastric cancer. *Cancer*, (in press).

GRIGIONI WF, D'ERRICO A, FORTUNATO C, FIORENTINO M, MANCINI AM, STETLER-STEVENSON WG, SOBEL ME, LIOTTA LA, ONISTO M AND GARBISA S. (1994). Prognosis of gastric carcinoma revealed by interactions between tumor cells and basement membrane. *Mod. Pathol.*, **7**, 220–225.

HANEMAAIJER R, KOOLWIJK P, LE CLERCQ L, DE VREE WJA AND VAN HINSBERGH VWM. (1993). Regulation of matrix metalloproteinase expression in human vein and microvascular endothelial cells. *Biochem. J.*, **296**, 803–809.

HASUI Y, MARUTSUKA K, SUZUMIYA J, KITADA S, OSADA Y AND SUMIYOSHI A. (1992). The content of urokinase-type plasminogen activator antigen as a prognostic factor in urinary bladder cancer. *Int. J. Cancer*, **50**, 871–873.

JÄNICKE F, SCHMITT M AND GRAEFF H. (1991). Clinical relevance of the urokinase-type and tissue-type plasminogen activators and of their type 1 inhibitor in breast cancer. *Semin. Thromb. Hemost.*, **17**, 303–312.

KAPLAN EL AND MEIER P. (1958). Nonparametric estimation from incomplete observations. *J. Am. Stat. Assoc.*, **53**, 457–481.

KIMURA T, IWAGAKI H, FUCHIMOTO S, HIZUTA A AND ORITA K. (1993). Relationship between type 1 collagenase activity, invasiveness and metastatic potential in colorectal carcinoma. *Cancer J.*, **6**, 77–80.

KLEINER DE AND STETLER-STEVENSON WG. (1994). Quantitative zymography: detection of picogram quantities of gelatinases. *Anal. Biochem.*, **218**, 325–329.

LOWRY OH, ROSEBROUGH NJ, FARR AL AND RANDALL RJ. (1951). Protein measurement with the folin phenol reagent. *J. Biol. Chem.*, **193**, 265–275.

MCDONNELL S, NAVRE M, COFFEY RJ AND MATRISIAN LM. (1991). Expression and localization of the matrix metalloproteinase Pump-1 (MMP-7) in human gastric and colon carcinomas. *Mol. Carcinogen.*, **4**, 527–533.

MATRISIAN LM. (1992). The matrix-degrading metalloproteinases. *BioEssays*, **14**, 455–463.

NAITO K, KANBAYASHI N, NAKAJIMA S, MURAI T, ARAKAWA K, NISHIMURA S AND OKUYAMA A. (1994). Inhibition of growth of human tumor cells in nude mice by a metalloproteinase inhibitor. *Int. J. Cancer*, **58**, 730–735.

NEKARDA H, SCHMITT M, ULM K, WENNINGER A, VOGELSANG H, BECKER K, RODER JD, FINK U AND SIEWERT JR. (1994). Prognostic impact of urokinase-type plasminogen activator and its inhibitor PAI-1 in completely resected gastric cancer. *Cancer Res.*, **54**, 2900–2907.

PEDERSEN H, BRÜNNER N, FRANCIS D, ØSTERLIND K, RØNNE E, HANSEN HH, DANØ K AND GRØNDAHL-HANSEN J. (1994). Prognostic impact of urokinase, urokinase receptor, and type 1 plasminogen activator inhibitor in squamous and large cell lung cancer tissue. *Cancer Res.*, **54**, 4671–4675.

POULSOM R, PIGNATELLI M, STETLER-STEVENSON WG, LIOTTA LA, WRIGHT PA, JEFFERY RE, LONGCROFT JM, ROGERS L AND STAMP GWH. (1992). Stromal expression of 72 kda Type IV collagenase (MMP-2) and TIMP-2 mRNAs in colorectal neoplasias. *Am. J. Pathol.*, **141**, 389–396.

SIER CFM, VERSPAGET HW, GRIFFIOEN G, VERHEIJEN JH, QUAX PHA, DOOIJEWAARD G, DE BRUIN PAF AND LAMERS CBHW. (1991). Imbalance of plasminogen activators and their inhibitors in human colorectal neoplasia. Implication of urokinase in colorectal carcinogenesis. *Gastroenterology*, **101**, 1522–1528.

WANG X, FU X, BROWN PD, CRIMMIN MJ AND HOFFMAN RM. (1994). Matrix metalloproteinase inhibitor BB-94 (Batimastat) inhibits human colon tumor growth and spread in a patient-like orthotopic model in nude mice. *Cancer Res.*, 4726–4728.

YAMAGATA S, YOSHII Y, SUH JG, TANAKA R AND SHIMIZU S. (1991). Occurrence of an active form of gelatinase in human gastric and colorectal carcinoma tissue. *Cancer Lett.*, **59**, 51–55.

ZUCKER S, LYSIK RM, ZARRABI MH AND MOLL U. (1993). Mr 92,000 collagenase is increased in plasma of patients with colon cancer and breast cancer. *Cancer Res.*, **53**, 140–146.

ZUCKER S, MANCUSO P, DIMASSIMO B, LYSIK RM, CONNER C AND WU CL. (1994). Comparison of techniques for measurement of gelatinases/type IV collagenases: enzyme-linked immunoassays versus substrate degradation assays. *Clin. Exp. Metast.*, **12**, 13–23.